# 353. New-Onset Diabetes as an Acute Complication of COVID-19: A National Population Cohort Analysis

**DOI:** 10.1093/ofid/ofab466.554

**Published:** 2021-12-04

**Authors:** Kwan Hong, Jeehyun Kim, Sujin Yum, Raquel Elizabeth Gómez Gómez, Byung Chul Chun

**Affiliations:** 1 Korea University College of Medicine, Seoul, Seoul-t’ukpyolsi, Republic of Korea; 2 Korea University, Seoul, Seoul-t’ukpyolsi, Republic of Korea

## Abstract

**Background:**

Diabetes is emerging as one of the complications of coronavirus disease 2019 (COVID-19), but this is hard to be revealed with cross-sectional studies since it is also known as the major predisposing factor for high-risk COVID-19. Therefore, this study aimed to estimate the risk of new-onset diabetes after COVID-19 through a population follow-up study.

**Methods:**

All COVID-19 confirmed cases in Korea from January 20 to June 4, 2020, were matched with national health insurance data and their health screening data, both provided by the National Health Insurance Service of Korea. Controls were selected as the people who received the PCR test for COVID-19 and showed negative results in the same period and followed up until July 19, 2020. We selected the outcome as the diagnosis of diabetes according to the 10th revision of the International Statistical Classification of Diseases and Related Health Problems (ICD-10, E10 – E14). People who were diagnosed with diabetes in the past five years were excluded from both groups. After performing a log-rank test between groups, adjusted incidence rate and hazard ratio were estimated using Cox proportional hazard modeling. Demographic characteristics (age, sex, region, family histories of hypertension/diabetes, and income) and underlying health conditions such as hypertension, dyslipidemia, heart disease, alcohol consumption, cigarette smoking, and BMI were adjusted. Proportional assumptions were tested by the zph test and the sensitivity analysis by excluding each factor in turn and comparing results.

**Results:**

A total of 6,247 COVID-19 patients and 143,594 controls without diabetes in the past were included for the analysis. The number of new-onset diabetes were 759 (12.15%) in COVID-19 patients and 3,465 (2.41%) in controls (P < 0.01). The adjusted incidence of diabetes was 15.34 (95% confidence interval, CI: 14.10 – 16.66) and 11.18 (95% CI: 10.67 – 11.72) per 100 person-year, respectively, with the mean follow-up time as 46.31 (standard deviation: 16.37) days. The adjusted hazard ratio of diabetes in COVID-19 cases was 2.97 (95% CI: 2.44 – 3.63).

**Conclusion:**

Since COVID-19 patients showed a higher incidence of new-onset diabetes in a short-time follow-up, we should consider diabetes as one of the possible complications of COVID-19.

**Disclosures:**

**All Authors**: No reported disclosures

